# Perinatal Outcomes During the COVID-19 Pandemic in Ontario, Canada

**DOI:** 10.1001/jamanetworkopen.2021.10104

**Published:** 2021-05-12

**Authors:** Andrea N. Simpson, John W. Snelgrove, Rinku Sutradhar, Karl Everett, Ning Liu, Nancy N. Baxter

**Affiliations:** 1 Department of Obstetrics and Gynaecology, St Michael’s Hospital, Unity Health Toronto, Toronto, Ontario, Canada; 2 Li Ka Shing Knowledge Institute, St Michael’s Hospital, Toronto, Ontario, Canada; 3 ICES, Toronto, Ontario, Canada; 4 Department of Obstetrics and Gynaecology, Sinai Health System, Toronto, Ontario, Canada; 5 Dalla Lana School of Public Health, Division of Biostatistics, University of Toronto, Toronto, Ontario, Canada; 6 Institute of Health Policy, Management and Evaluation, University of Toronto, Toronto, Ontario, Canada; 7 Department of Surgery, University of Toronto, Toronto, Ontario, Canada; 8 Melbourne School of Population and Global Health, University of Melbourne, Melbourne, Victoria, Australia

## Abstract

This cohort study evaluates rates of preterm birth and stillbirth in Ontario, Canada, during the first 6 months of the COVID-19 pandemic.

## Introduction

Public health measures to control the COVID-19 pandemic may be associated with reduced risk of preterm birth (PTB).^1,2^ Conversely, avoidance of health care may be associated with increased risk of stillbirth.^3^ We evaluated rates of PTB and stillbirth during the first 6 months of the pandemic because previous studies conducted early in the pandemic have had inconsistent results.

## Methods

We performed a population-based cohort study in Ontario, Canada, using linked databases at ICES (formerly Institute for Clinical Evaluative Sciences).^4^ Data use without consent is authorized under section 45 of Ontario’s Personal Health Information Protection Act; thus, review by a research ethics board was not required. We followed the Strengthening the Reporting of Observational Studies in Epidemiology (STROBE) reporting in epidemiology guideline.^5^

In-hospital births at 20 weeks’ or more gestational age (GA) from March 15 to September 30, 2020 (pandemic group), were compared with corresponding calendar periods from 2015 to 2019 (historical group) (eFigure in the Supplement). Births were identified in the Mother-Baby Data Set derived from the Canadian Institute for Health Information Discharge Abstract Database. Maternal characteristics included age, parity, singleton vs multiple gestation, area-level income quintile, comorbidities, pregnancy conceived with assisted reproductive technology, and SARS-CoV-2 infection during pregnancy.

PTB (live birth at <37 weeks’ GA) and stillbirths (intrauterine death at ≥20 weeks’ GA) were the primary outcomes. Secondary outcomes were extreme PTB (<28 weeks’ GA), very PTB (<32 weeks’ GA), severe small for GA (birth weight less than the fifth percentile for sex and GA), neonatal intensive care unit admission, and early (up to 7 days) and late (8-28 days) neonatal death.

We used univariable and multivariable logistic regression models to examine the association between birth period (pandemic vs historical) and odds of each outcome. A generalized estimating equations approach was used to account for clustering at the level of birth institution. We assessed the effect of time spent in the pandemic by incorporating an interaction term between our exposure (pandemic vs historical birth) and number of weeks since March 15, 2020, for each PTB outcome. Analyses were performed using SAS Enterprise Guide statistical software version 7.15 (SAS Institute). Statistical tests were 2-sided, with α < .05 considered significant. Data analysis was performed from March 15 to September 30, 2020.

## Results

A total of 67 747 births occurred during the pandemic period, and 348 633 births occurred during the historical period. There were no differences in baseline characteristics between groups. There was no difference in the proportion of PTBs (5103 [7.5%] vs 26 216 [7.5%] PTBs) or stillbirths (347 [0.5%] vs 1799 [0.5%] stillbirths) between the pandemic and historical groups. After multivariable analysis, the adjusted odds ratio (aOR) for PTB was 1.00 (95% CI, 0.97-1.03), and that for stillbirth was 0.99 (95% CI, 0.89-1.11) (Table). We observed a small but significant difference in very PTB (<32 weeks’ GA) in the 2 groups (4531 [1.3%] vs 807 [1.2%] very PTBs; OR, 0.89; 95% CI, 0.80-0.99), which persisted after multivariable adjustment (aOR, 0.91; 95% CI, 0.85-0.98). There were no differences in extreme PTB, severe small for GA, neonatal intensive care unit admission, or neonatal death. We found no significant association between time spent in the pandemic and any outcome.

**Table.** Multivariable Generalized Estimating Equation Logistic Regression Analyses to Determine the Odds of Adverse Perinatal Outcomes During the Pandemic Period Compared With the Historical Period

Outcome	Births, No. (%)		OR (95% CI)	
	Pandemic group (n = 67 747)	Historical group (n = 348 633)	Unadjusted	Adjusted^a^
Preterm birth (<37 wk GA)	5103 (7.5)	26 216 (7.5)	1.00 (0.95-1.04)	0.99 (0.97-1.03)
Stillbirth	347 (0.5)	1799 (0.5)	0.99 (0.89-1.11)	0.99 (0.89-1.11)
Extreme preterm birth (<28 wk GA)	406 (0.6)	2254 (0.6)	0.90 (0.78-1.04)	0.91 (0.80-1.03)
Very preterm birth (<32 wk GA)	807 (1.2)	4531 (1.3)	0.89 (0.80-0.99)^b^	0.91 (0.85-0.98)^b^
Severe small for GA	3826 (5.6)	19 225 (5.5)	1.02 (0.98-1.07)	1.02 (0.98-1.08)
Neonatal intensive care unit admission	8526 (12.6)	43 049 (12.3)	1.01 (0.93-1.10)	1.01 (0.94-1.08)
Neonatal death				
Early	83 (0.1)	539 (0.2)	0.75 (0.50-1.14)	0.77 (0.51-1.15)
Late	30 (0.0)	165 (0.0)	0.91 (0.52-1.60)	0.96 (0.61-1.51)

Abbreviations: GA, gestational age; OR, odds ratio.

^a^ Adjusted analysis included the following variables: maternal age at index birth (continuous), parity (number of births ≥20 weeks’ GA, continuous), singleton vs multiple birth (binary), Aggregated Diagnosis Groups score (continuous), income quintile (categorical, with 1 as the lowest and 5 as the highest), rural residence (binary, urban vs rural), preexisting hypertension (binary), preexisting diabetes (binary), pregnancy conceived with assisted reproductive technology (binary), short interbirth interval (<18 months, binary), and history of preterm birth (binary).

^b^ Denotes significance. Observations with missing variables were excluded from the model.

## Discussion

We found no differences in the overall risk of PTB, stillbirth, or other perinatal outcomes during the first 6 months of the COVID-19 pandemic. We observed a small reduction in PTB at less than 32 weeks’ GA, similar to Denmark and Ireland, where comparable strict lockdown measures were in effect.^1,2^ In contrast, no difference in PTB was observed in a population-based study in Sweden, where strict lockdown orders were not in effect.^6^

Limitations of this study include the inability to evaluate out-of-hospital births; however, less than 3% of births in Ontario occur outside of hospitals. We could not evaluate for some factors that influence PTB risk, such as smoking. We did not evaluate the risk of PTB among women who experienced COVID-19 during pregnancy because this number was small.

The COVID-19 pandemic first wave did not coincide with significant changes in overall PTB or stillbirth in Ontario. A small reduction in PTB at less than 32 weeks’ GA suggests that strict lockdown measures may have been associated with reduced risk in this subgroup.

## Notes

Supplement. **eFigure.** Flow Diagram of Included Births in the Historical and Pandemic Groups
