# Supplementary material for: Perinatal Outcomes During the COVID-19 Pandemic in Ontario, Canada
Source: JAMA Netw Open. 2021 May 12;4(5):e2110104. doi: 10.1001/jamanetworkopen.2021.10104 (PMC8116980; doi:10.1001/jamanetworkopen.2021.10104)
Supplement: Supplement. — eFigure. Flow Diagram of Included Births in the Historical and Pandemic Groups [file jamanetwopen-e2110104-s001.pdf]

Supplemental Online Content

Simpson AN, Snelgrove JW, Sutradhar R, Everett K, Liu N, Baxter NN. Perinatal outcomes during the COVID-19 pandemic in Ontario, Canada. *JAMA Netw Open*. 2021;4(5):e2110104. doi:10.1001/jamanetworkopen.2021.10104

eFigure. Flow Diagram of Included Births in the Historical and Pandemic Groups

This supplemental material has been provided by the authors to give readers additional information about their work.

© 2021 Simpson AN et al. *JAMA Network Open.*

eFigure 1. Flow Diagram of Included Births in the Historical and Pandemic Groups

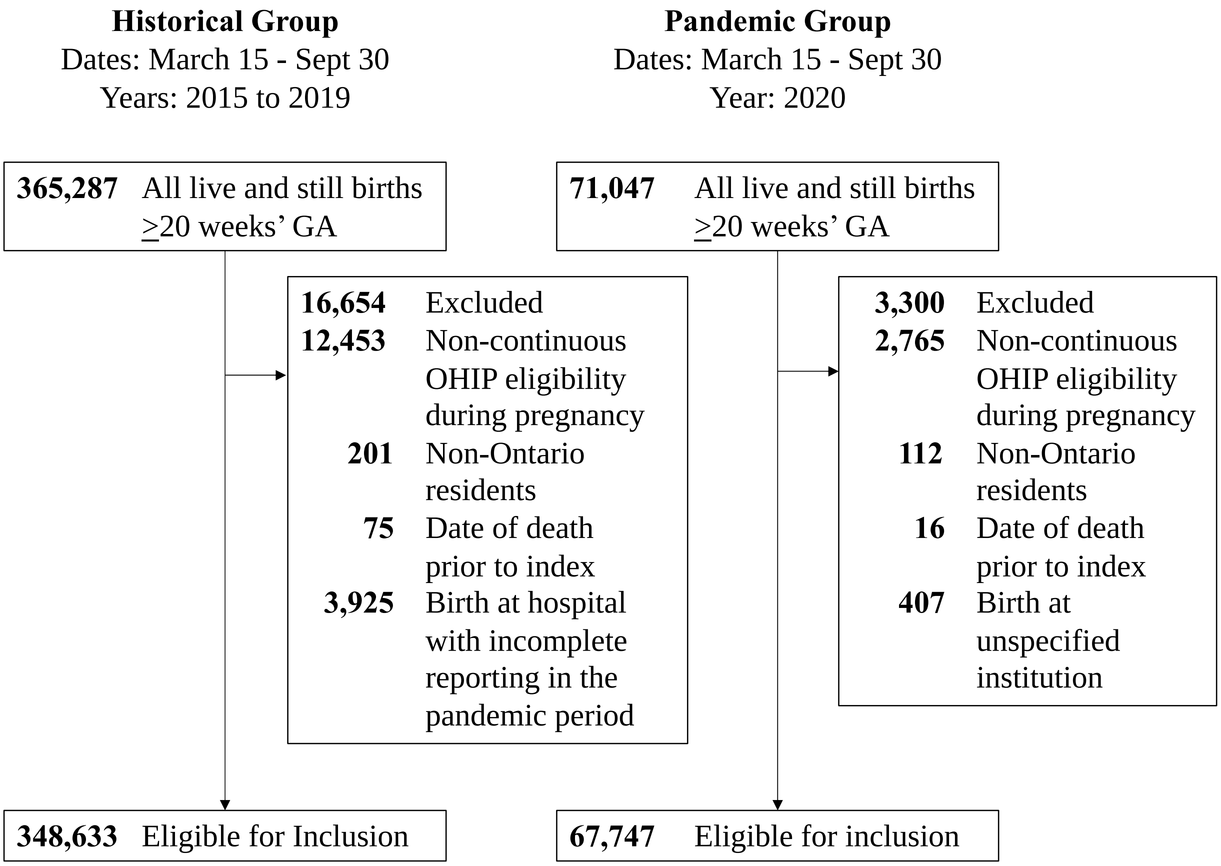

© 2021 Simpson AN et al. *JAMA Network Open.*
